# Testicular prostheses implantation in cisgender and transgender males

**DOI:** 10.1007/s00345-026-06916-x

**Published:** 2026-11-14

**Authors:** Navid Roessler, Malte W. Vetterlein, Felix Lübbersmeyer, Dennis Thölke, Tim A. Ludwig, Roland Dahlem, Margit Fisch, Florian Janisch

**Affiliations:** Department of Urology, University Medical Center Hamburg-Eppendorf, Martinistraße 52, 20246 Hamburg, Germany

**Keywords:** Testicular prostheses, Patient-reported outcome measures, Quality of life, Prosthesis, Gender-affirming surgery

## Abstract

**Objectives:**

To evaluate clinical and patient-reported outcome measures (PROMs) following testicular prostheses (TP) implantation in cisgender and transgender males.

**Methods:**

Patients undergoing TP implantation between January 2013 and January 2026 were retrospectively identified and stratified by self-reported gender identity. Retreatment-free survival (RFS) was estimated using the Kaplan–Meier method and compared between groups using the log-rank test. PROMs included the ICIQ-Satisfaction (ICIQ-S) outcome score and satisfaction with surgery item, the Decision Regret Scale (DRS), and the Male Genital Self-Image Scale (MGSIS).

**Results:**

A total of 91 patients were included, comprising 48 cisgender (53%) and 43 transgender males (47%). Within a median follow-up of 43 months (95% CI 29–56), surgical retreatment was recorded in four cisgender (8.3%) and 13 transgender males (30%). There was a statistically significant higher retreatment rate in transgender males (log-rank p = 0.004), with RFS at 12 and 24 months of 92% at both time points in cisgender versus 70% and 64% in transgender males. Median ICIQ-S outcome scores were 21 (IQR 17–22) in cisgender and 21 (IQR 19–23) in transgender males (p = 0.2), and median ICIQ-S satisfaction with surgery item was 9 (IQR 8–10) in both groups (p > 0.9). Decision regret was low in both groups, with a median DRS of 5 (IQR 0–17.5) in cisgender and 0 (IQR 0–5) in transgender males (p = 0.039). Median MGSIS was 22 (IQR 21–26) in cisgender and 22 (IQR 20–26) in transgender males (p = 0.2). Limitations include the retrospective single-center design.

**Conclusion:**

Transgender males demonstrate significantly lower RFS compared with cisgender males, largely reflecting planned staged procedures within gender-affirming surgery rather than TP-related failure. Patient-reported satisfaction and genital self-image are high and comparable across both groups, with low overall decision regret, supporting TP implantation in both cisgender and transgender males.

**Supplementary Information:**

The online version contains supplementary material available at https://doi.org/10.1007/s00345-026-06916-x.

## Introduction

Testicular prostheses (TP) implantation is a well-established intervention for male patients experiencing unilateral or bilateral testicular loss due to orchiectomy, congenital absence, trauma, or in gender-affirming surgery [1, 2]. Beyond its functional and aesthetic purpose, TP implantation addresses profound psychological consequences affecting body image, self-esteem, and sense of bodily completeness, thereby contributing meaningfully to psychological well-being and overall quality of life in affected patients [3, 4].

The implantation of TP has grown in recent years, driven by the expanding role of gender-affirming surgery in transgender males [5]. Transgender males undergoing scrotoplasty with TP implantation represent a distinct population, in whom neoscrotal tissue characteristics, prior surgical history, and the complexity of staged procedures including subsequent penile prosthesis implantation may all contribute to higher rates of complications and reintervention compared to cisgender males. Yet robust comparative data addressing whether reintervention rates and patient reported quality of life differ remain scarce.

Contemporary evidence on TP outcomes is further limited by a lack of methodological standardization [6]. Patient-reported outcomes are frequently assessed using heterogeneous, non-validated tools, rendering cross-study comparisons difficult and evidence-based counselling poorly supported [7–10]. The absence of validated, standardized patient-reported outcome measures (PROMs) is particularly consequential in a field where the primary goal of surgery is patient well-being rather than a measurable clinical endpoint. Establishing a reliable and comparable evidence base is therefore essential to improve preoperative counselling and to better align patient expectations with achievable outcomes.

The present study evaluates surgical and patient-reported outcomes in a comparative cohort of cisgender and transgender male patients who underwent TP implantation at our institution. By applying validated PROMs and standardized outcome reporting, this study aims to provide population-specific evidence to support individualized preoperative counselling, optimize patient satisfaction, and advance standardized outcome reporting in TP surgery.

## Methods

The study protocol was approved by the Institutional Review Board (2021-100628-BO-ff).

### Study population

This study included all patients undergoing TP implantation at our institution between beginning of January 2013 and end of January 2026. Gender identity was based on patient self-identification, with patients subsequently categorized into cisgender and transgender male cohorts for analysis. Patients undergoing isolated explantation or surgical revision of an externally implanted TP were excluded due to missing perioperative data. Active smoking was not an exclusion criterion. Perioperative and follow-up morbidity data were prospectively collected from electronic medical records (Soarian Clinicals). Data extraction from electronic medical records was performed systematically and transferred into a dedicated database. Follow-up was conducted by identifying patients through the institutional database and contacting them via structured telephone interviews performed by medical staff (DT, FL). In addition, consenting patients received an email containing a REDCap (Research Electronic Data Capture) survey link to complete standardized questionnaires at a single follow-up time point. Patients without a TP in situ at the time of survey completion were instructed to respond based on their experience during the period of first implantation. All follow-up data were subsequently integrated into the same database. Follow-up was defined as the interval from TP implantation to the last contact, either in person or by telephone.

### Clinical and surgical characteristics

Preoperatively, patients underwent a standardized clinical assessment during which comorbidities and clinical characteristics were systematically documented. Preoperative data included patient demographics (e.g., age, body mass index, smoking status) and comorbidities (e.g., hypertension, hyperlipidaemia, diabetes mellitus) and oncological history where applicable. The overall comorbidity burden was additionally quantified using the age-adjusted Charlson Comorbidity Index (CCI) [11]. In addition, detailed clinical history was recorded, including previous TP implantation. In cisgender males presenting with testicular malignancy, simultaneous TP implantation was routinely offered prior to radical orchiectomy. In transgender males, TP implantation was performed as part of a staged gender-affirming surgical approach, as concomitant penile prosthesis and TP implantation was not performed within the same operative setting.

As an institutional standard for prosthetic surgery at our center, patients generally received intravenous cefuroxime (1500 mg three times daily) and gentamicin (240 mg once daily) for approximately three days postoperatively; discharge was individualized and occasionally occurred earlier. Intraoperative data comprised the American Society of Anesthesiologists (ASA) physical status classification. All implanted testicular prostheses were pre-filled devices manufactured by POLYTECH Health & Aesthetics (Dieburg, Germany, silicone gel-filled / soft silicone elastomer), available in sizes S, M and L (S, 12 mL; M, 18 mL; L, 22 mL). During preoperative counselling, all patients were shown sizer models and asked about their preferred size. In cisgender males, size was chosen by comparison with the contralateral testis. In transgender males, patients were explicitly informed that the final size depends on intraoperative findings, particularly the available neoscrotal tissue, and was determined accordingly. Postoperative complications within 30 days were recorded and graded according to the validated and adapted Clavien-Dindo Classification (CDC) [12].

### Study endpoints

The primary objective outcome was retreatment-free survival (RFS), defined as the time from TP implantation to any surgical reintervention, with patients free of retreatment censored at last follow-up. Retreatment included planned explantations within staged gender-affirming surgery (to allow penile prosthesis pump placement) as well as unplanned retreatments; reasons were recorded separately.

Subjective outcomes were assessed using a set of validated PROMs, comprising the ICIQ-Satisfaction (ICIQ-S) outcome score and satisfaction with surgery item [13], the Decision Regret Scale (DRS) [14], and the Male Genital Self-Image Scale (MGSIS) [15]. In addition, a structured TP-specific questionnaire was administered to capture patient-perceived TP characteristics including size, weight, consistency, shape, position, and comfort, each rated on a five-point Likert scale, as well as patient-related contextual factors including sociodemographic and clinical characteristics.

### Statistical analysis

Baseline clinical characteristics were analyzed using descriptive statistics. Categorical variables are presented as absolute counts and percentages, while continuous variables are presented as medians with interquartile ranges (IQRs). Between-group comparisons were performed using the Mann–Whitney U test for continuous variables and Fisher's exact test for categorical variables. RFS was estimated using the Kaplan–Meier method, with median follow-up time calculated using the reverse Kaplan–Meier method [16]. Survival curves were compared between cisgender and transgender males using the log-rank test, and numbers at risk were displayed at predefined time points.

PROMs were evaluated according to their respective scoring manuals. Surgical outcome satisfaction was evaluated using the ICIQ-S, comprising the outcome score (six items, total score 0–24, higher scores indicating greater satisfaction) and the satisfaction with surgery item (single item, score 0–10, higher scores indicating greater satisfaction). Decision regret was quantified using the DRS, a five-item instrument yielding a total score from 0 to 100, where higher scores reflect greater regret. Genital self-image was assessed using the MGSIS, a seven-item instrument with a total score ranging from 7 to 28, where higher scores indicate a more positive genital self-image. PROM scores are reported as medians with IQRs, and score distributions were visualized using violin plots. All statistical analyses were performed using Stata version 19 (StataCorp, College Station, TX, USA). A two-sided p-value of less than 0.05 was considered statistically significant.

## Results

A total of 91 patients were included in the final analysis, comprising 48 cisgender males (53%) and 43 transgender males (47%).

### Descriptive analysis of clinical and surgical characteristics

The demographic and clinical characteristics of the study cohort are summarized in Table 1. Median age at implantation was 34 years (IQR 27–41) in cisgender and 33 years (IQR 28–44) in transgender males (p = 0.4).

**Table 1:** Baseline and perioperative characteristics of cisgender and transgender males undergoing testicular prostheses implantation

Variable	Total (N = 91)	Cisgender (N = 48)	Transgender (N = 43)	p-value
Age at implantation (yr), median (IQR)				0.4
	33 (28–43)	34 (27–41)	33 (28–44)	
BMI (kg/m^2^), median (IQR)				0.9
	25 (23–29)	25 (23–29)	25 (23–28)	
Indication for TP implantation, N (%)				< 0.001
Testicular cancer	37 (41)	37 (77)	0 (0)	
Gender-affirming surgery	43 (47)	0 (0)	43 (100)	
Torsion	4 (4.4)	4 (8.3)	0 (0)	
Trauma	1 (1.1)	1 (2.1)	0 (0)	
Congenital	6 (6.6)	6 (13)	0 (0)	
Smoking status, N (%)				0.6
Never	52 (57)	30 (63)	22 (51)	
Former	2 (2.2)	1 (2.1)	1 (2.3)	
Active	37 (41)	17 (35)	20 (47)	
Comorbidities, N (%)				
Diabetes mellitus	2 (2.2)	0 (0)	2 (4.7)	0.2
Hyperlipidaemia	9 (9.9)	3 (6.2)	6 (14)	0.3
Hypertension	16 (18)	5 (10)	11 (26)	0.1
Lung disease	9 (9.9)	3 (6.2)	6 (14)	0.3
Neuropathy	3 (3.3)	1 (2.1)	2 (4.7)	0.6
Age-adjusted CCI, N (%)				0.001
CCI ≤ 2	74 (81)	33 (69)	41 (96)	
CCI 3–5	6 (6.6)	4 (8.3)	2 (4.7)	
CCI ≥ 6	11 (12)	11 (23)	0 (0)	
ASA status, N (%)				0.8
ASA I	28 (31)	15 (31)	13 (30)	
ASA II	57 (63)	29 (60)	28 (65)	
ASA III	6 (6.6)	4 (8.3)	2 (4.7)	
Prior TP implantation, N (%)				0.004
None	84 (92)	48 (100)	36 (84)	
One	5 (5.5)	0 (0)	5 (12)	
Two	2 (2.2)	0 (0)	2 (4.7)	
Number of TP implanted, N (%)				< 0.001
One	51 (56)	44 (92)	7 (16)	
Two	40 (44)	4 (8.3)	36 (84)	
Implantation site, N (%)				< 0.001
Left	22 (24)	17 (35)	5 (12)	
Right	29 (32)	27 (56)	2 (4.7)	
Bilateral	40 (44)	4 (8.3)	36 (84)	
TP size, N (%)				0.007
S	7 (7.7)	1 (2.1)	6 (14)	
M	80 (88)	47 (98)	33 (77)	
S + M	3 (3.3)	0 (0)	3 (7)	
M + L	1 (1.1)	0 (0)	1 (2.3)	
Length of stay (days), median (IQR)				< 0.001
	4 (3–6)	3 (2–3)	6 (4–6)	

Percentages may not add up to 100%, as they are rounded

*yr* years, *IQR* interquartile range, *BMI* body mass index, *CCI* Charlson comorbidity index, ASA American society of anesthesiologists, TP testicular prostheses

The predominant reason for TP implantation in cisgender males was testicular loss due to orchiectomy for testicular cancer (N = 37, 77%), while the remaining cases were attributable to testicular torsion (N = 4, 8.3%), trauma (N = 1, 2.1%), and congenital or developmental conditions (N = 6, 13%). All transgender males underwent TP implantation as part of a staged gender-affirming surgical approach (N = 43, 100%).

Smoking status was comparable between groups (p = 0.6), with active smoking reported in 17 cisgender (35%) and 20 transgender males (47%). Comorbidities were generally infrequent in both cohorts; hypertension was the most prevalent condition, present in 11 transgender (26%) compared to five cisgender males (10%; p = 0.1). Most patients were classified as CCI ≤ 2 (cisgender: N = 33, 69%; transgender: N = 41, 96%), while CCI ≥ 6 was observed only in cisgender males (N = 11, 23%).

ASA classification was similar between groups (p = 0.8), with ASA II predominating in both cisgender (N = 29, 60%) and transgender males (N = 28, 65%). Prior TP implantation was recorded in seven transgender males (16%) compared to none in the cisgender cohort (p = 0.004). Bilateral implantation was performed in 36 transgender (84%) compared to four cisgender males (8.3%; p < 0.001), with TP size M being the most frequently implanted in both groups (cisgender: N = 47, 98%; transgender: N = 33, 77%). Median hospital stay was longer in transgender males (6 days, IQR 4–6) compared to cisgender males (3 days, IQR 2–3; p < 0.001). Within 30 days of surgery, complications were infrequent and comparable between groups. Grade 1 complications (conservatively managed hematoma) occurred in four patients (4.4%), one patient required suprapubic catheterization for urinary retention (CDC 3 A), and three patients underwent surgical hematoma evacuation (CDC 3 B; cisgender: N = 1, transgender: N = 2).

### Retreatment outcomes and Kaplan–Meier analysis

Within a median follow-up of 43 months (95% CI 29–56), retreatment was recorded in 17 patients (19%), comprising four cisgender (8.3%) and 13 transgender males (30%).

In cisgender males, retreatment consisted of explantation in three patients and TP exchange in one, with indications including infection (N = 2), inguinal dislocation (N = 1), and TP defect (N = 1). In transgender males, retreatment consisted of unilateral explantation in six patients, bilateral explantation in five, and TP exchange in two, with indications comprising infection (N = 2), scrotal erosion (N = 2), cosmetic dissatisfaction (N = 3), and explantation to facilitate ipsilateral penile prosthesis pump placement (N = 6). These planned explantations are not complications; excluding them, retreatment occurred in 4 cisgender (8.3%) and 7 transgender males (16%).

**Fig. 1:**
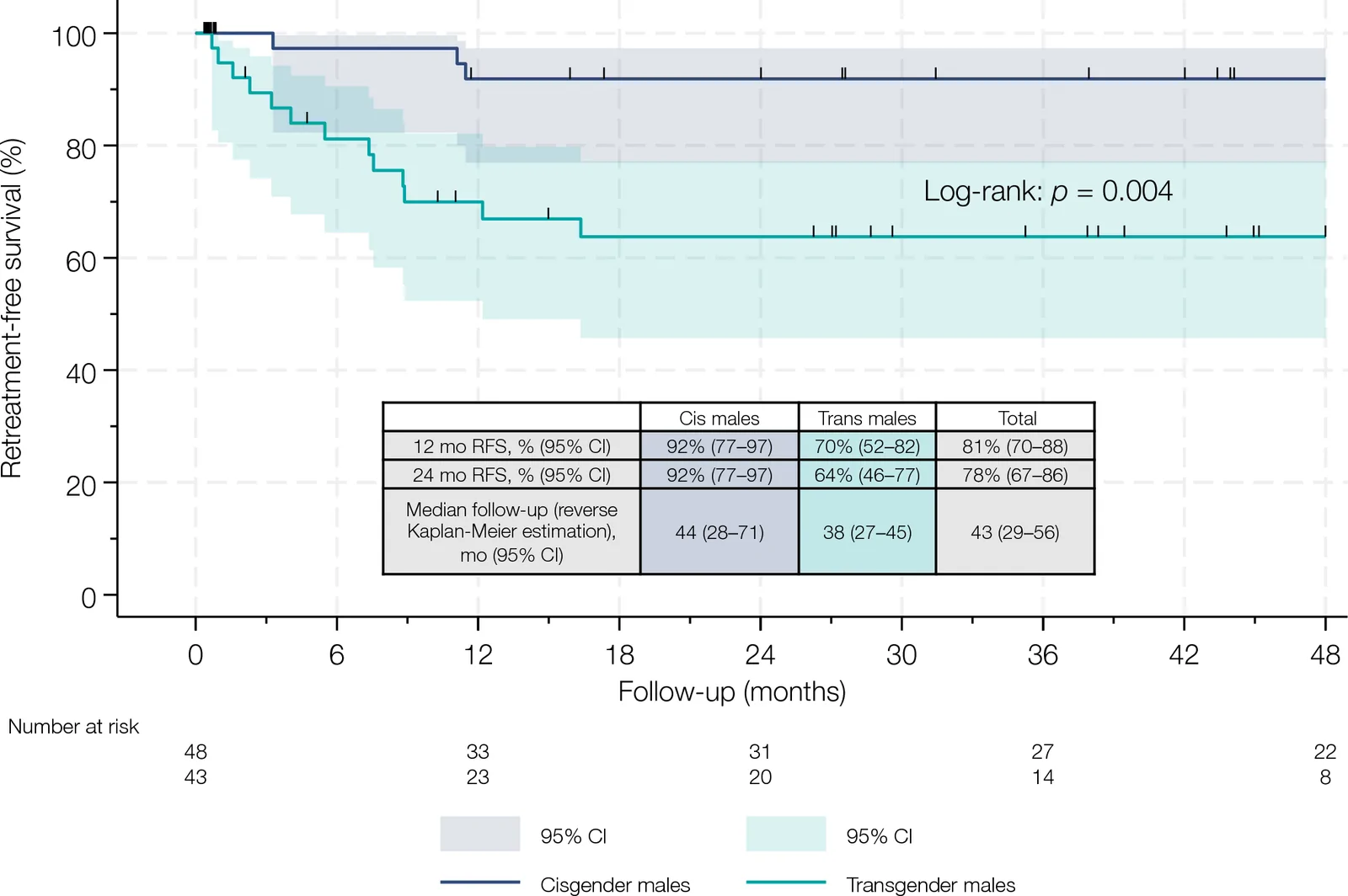
Kaplan–Meier estimates of retreatment-free survival following first testicular implantation, stratified by gender (cisgender male vs. transgender male) *CI* confidence interval, *RFS* retreatment-free survival

There was a statistically significant difference in RFS between cisgender and transgender males (log-rank p = 0.004), with RFS at 12 and 24 months of 92% (95% CI 77–97) at both time points in cisgender males, compared to 70% (95% CI 52–82) and 64% (95% CI 46–77) in transgender males, respectively (Fig. 1). RFS remained stable in cisgender males between 12 and 24 months, whereas it continued to decline in transgender males.

### Patient-reported outcome measures

**Fig. 2:**
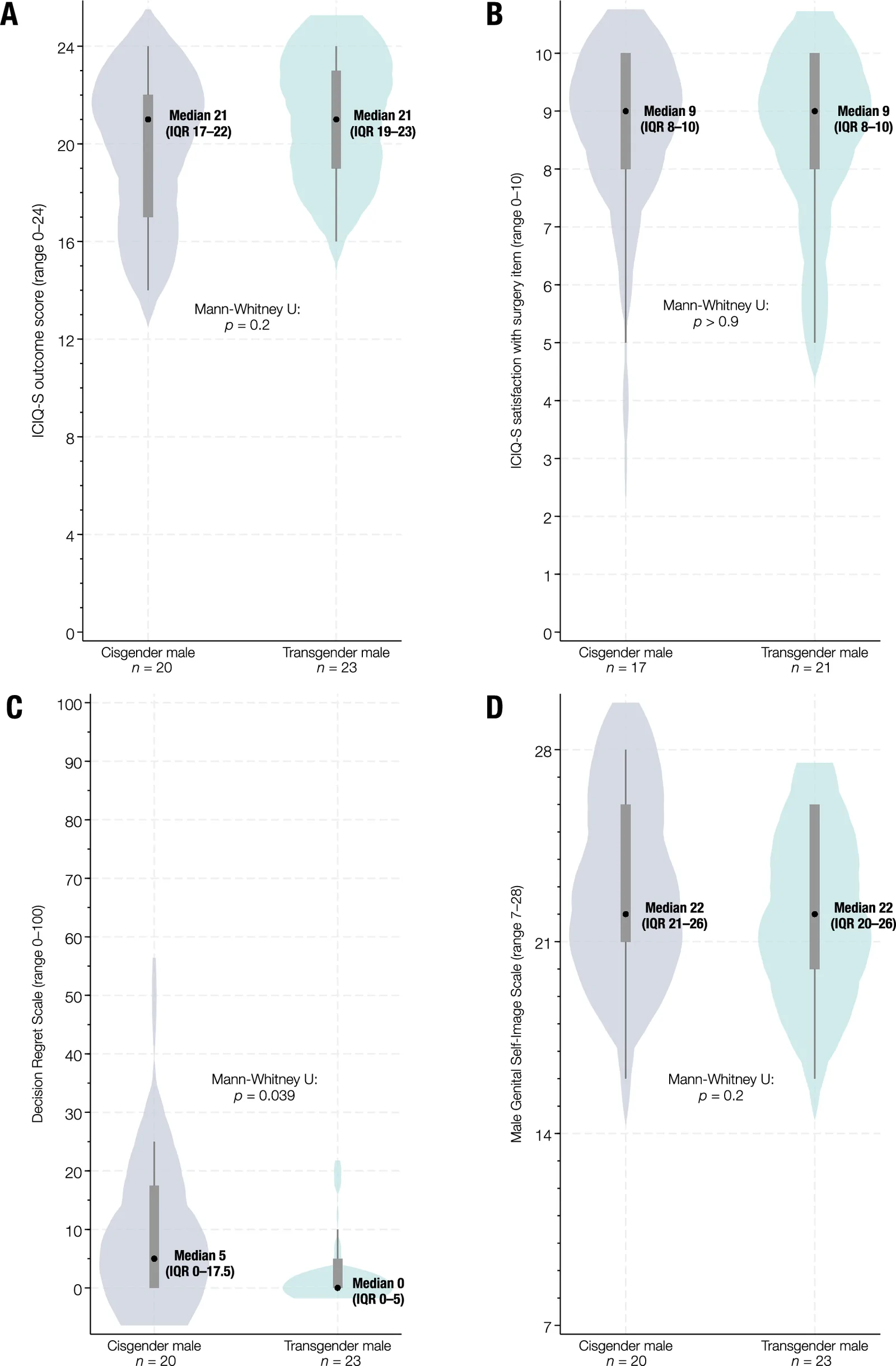
Violin plots illustrating the distribution of patient-reported outcome measures (PROMs) scores stratified by gender (cisgender male vs. transgender male). Panels depict: **A** ICIQ-S outcome score, **B** ICIQ-S satisfaction with surgery item, **C** Decision Regret Scale, and **D** Male Genital Self-Image Scale

PROM results are illustrated in Fig. 2, and detailed response distributions are provided in Supplementary File 1–3. Of 91 patients, 64 (70%) were successfully contacted by telephone, of whom 43 (67%) completed and returned the PROMs questionnaire (cisgender: N = 20, transgender: N = 23). At the time of survey completion, all responding patients had TP in situ.

The median ICIQ-S outcome score was 21 (IQR 17–22) in cisgender and 21 (IQR 19–23) in transgender males, with no statistically significant difference between groups (p = 0.2, Fig. 2A). The median ICIQ-S satisfaction with surgery item was 9 (IQR 8–10) in both groups (p > 0.9, Fig. 2B), reflecting high surgical satisfaction across both cohorts. Decision regret was low overall; the median DRS score was 5 (IQR 0–17.5) in cisgender compared to 0 (IQR 0–5) in transgender males, with cisgender males demonstrating significantly higher regret scores (p = 0.039, Fig. 2C). Genital self-image was comparable between groups, with a median MGSIS score of 22 (IQR 21–26) in cisgender and 22 (IQR 20–26) in transgender males (p = 0.2, Fig. 2D).

Regarding TP-specific characteristics, most patients rated TP weight (N = 37, 86%), shape (N = 35, 81%), position (N = 35, 81%), and comfort (N = 19, 44%) as appropriate. TP size was rated as appropriate by 15 cisgender (75%) and 12 transgender males (52%), with a tendency toward perceiving the TP as too small in transgender males (44% vs. 25%; p = 0.2). TP consistency demonstrated a statistically significant between-group difference, rated as appropriate by 50% (N = 10) of cisgender compared to 87% (N = 20) of transgender males, with cisgender males more frequently reporting the TP as too firm (40% vs. 8.7%; p = 0.008). Detailed response distributions are provided in Table 2.

**Table 2:** Patient-reported prostheses-specific characteristics among cisgender and transgender males completing the patient-reported outcome measures (PROMs) questionnaire

Variable	Total (N = 43)	Cisgender (N = 20)	Transgender (N = 23)	p-value
TP size, N (%)				0.2
Much too large	0 (0)	0 (0)	0 (0)	
Too large	0 (0)	0 (0)	0 (0)	
Appropriate	27 (63)	15 (75)	12 (52)	
Too small	15 (35)	5 (25)	10 (44)	
Much too small	0 (0)	0 (0)	0 (0)	
Not reported	1 (2.3)	0 (0)	1 (4.3)	
TP weight, N (%)				0.055
Much too heavy	0 (0)	0 (0)	0 (0)	
Too heavy	2 (4.7)	2 (10)	0 (0)	
Appropriate	37 (86)	18 (90)	19 (83)	
Too light	4 (9.3)	0 (0)	4 (17)	
Much too light	0 (0)	0 (0)	0 (0)	
TP consistency, N (%)				0.008
Much too firm	2 (4.7)	2 (10)	0 (0)	
Too firm	10 (23)	8 (40)	2 (8.7)	
Appropriate	30 (70)	10 (50)	20 (87)	
Too soft	1 (2.3)	0 (0)	1 (4.3)	
Much too soft	0 (0)	0 (0)	0 (0)	
TP shape, N (%)				0.2
Not fitting at all	1 (2.3)	0 (0)	1 (4.3)	
Rather not fitting	3 (7)	3 (15)	0 (0)	
Appropriate	35 (81)	16 (80)	19 (83)	
Rather well fitting	2 (4.7)	1 (5)	1 (4.3)	
Very well fitting	2 (4.7)	0 (0)	2 (8.7)	
TP position, N (%)				0.3
Much too high	1 (2.3)	0 (0)	1 (4.3)	
Too high	3 (7)	3 (15)	0 (0)	
Appropriate	35 (81)	16 (80)	19 (83)	
Too low	2 (4.7)	1 (5)	1 (4.3)	
Much too low	2 (4.7)	0 (0)	2 (8.7)	
TP comfort, N (%)				0.2
Very uncomfortable	0 (0)	0 (0)	0 (0)	
Rather uncomfortable	2 (4.7)	2 (10)	0 (0)	
Appropriate	19 (44)	8 (40)	11 (48)	
Rather comfortable	6 (14)	1 (5)	5 (22)	
Very comfortable	16 (37)	9 (45)	7 (30)	

Percentages may not add up to 100%, as they are rounded

*TP* testicular prostheses

Regarding patient-related contextual factors, most patients in both groups considered being offered a TP as very important (cisgender: N = 16, 80%; transgender: N = 18, 78%; p = 0.9). The feeling of completeness was the predominant reason for choosing implantation overall (N = 38, 88%), followed by aesthetic reasons (N = 23, 54%) and improved body image (N = 20, 47%). Detailed response distributions are provided in Supplementary File 4.

## Discussion

In this comparative cohort study of patients undergoing TP implantation, transgender males demonstrated a lower RFS compared to cisgender males, predominantly driven by planned subsequent procedures within staged gender-affirming surgery rather than TP-related complications. As the analysis was based on all-cause retreatment, planned explantations are included and should not be interpreted as complications. Patients should be counselled that a TP in transgender males has a higher likelihood of being removed or exchanged over time, for both planned and unplanned reasons. Despite this difference in retreatment patterns, patient-reported satisfaction and genital self-image remained consistently high and comparable between groups, with low decision regret observed in both cohorts.

### Retreatment and perioperative management

The higher retreatment rate observed in transgender males was predominantly driven by explantation in preparation for subsequent penile prosthesis implantation, reflecting an expected step within staged gender-affirming reconstruction rather than device failure per se. Neoscrotal tissue characteristics and prior reconstructive surgery may limit immediate accommodation of prostheses components [17, 18], thereby necessitating a sequential approach in which TP implantation is performed according to institutional standards to allow tissue adaptation and pocket formation prior to further reconstructive steps and to optimize anatomical conditions for later penile prosthesis pump implantation. The hospital stay in our cohort reflects institutional practice rather than medical necessity, including a standardized intravenous antibiotic course of usually three days after prosthetic surgery. We acknowledge that testicular prosthesis implantation is performed safely as an outpatient or same-day procedure in other centers, and our length-of-stay data should be interpreted in this context. Despite differences in surgical sequencing, PROMs remained highly favourable, and the overall impact on quality of life appears substantial in both cisgender and transgender males, suggesting that TP implantation should be considered a beneficial intervention across both populations. Preoperative counselling should therefore emphasize both the low overall complication burden and the likelihood of additional planned procedures in transgender patients as part of a reconstructive pathway, rather than an indicator of treatment failure.

### Patient-reported satisfaction and decision regret

The median ICIQ-S outcome score and satisfaction with surgery item were similarly high in both cisgender and transgender males, indicating consistently favourable patient-reported outcomes across groups. This is particularly noteworthy given the disparity in retreatment rates, highlighting a dissociation between objective surgical burden and subjective satisfaction and underscoring the value of validated PROMs in assessing patient-centered outcomes. Decision regret was low in both groups, although cisgender males demonstrated higher regret scores than transgender males. While modest and of limited clinical relevance, this difference may reflect distinct motivational contexts underlying each group’s decision for implantation. In cisgender males, TP implantation typically follows testicular loss due to malignancy, with the decision made shortly after the oncological diagnosis, which may introduce residual uncertainty or ambivalence persisting at follow-up. In contrast, transgender males typically undergo implantation as an anticipated step within a staged gender-affirming surgical pathway, where the TP fulfills a pre-existing goal, further supported by feeling of completeness as the predominant motivation for implantation. Lower decision regret and improved body image following TP placement compared with non-implantation have been described in patients undergoing orchiectomy for testicular cancer, supporting its role in preoperative counselling [10]. Systematic application of the DRS in future studies may further clarify regret trajectories over time.

Genital self-image, assessed using the MGSIS, was similarly high in both groups. At the item level, dissatisfaction was mainly related to TP size, particularly among transgender males, who more frequently perceived the implant as too small compared with cisgender males. This is consistent with the TP-specific questionnaire. In both groups, patients were shown sizer models during preoperative counselling and indicated their preferred size. In cisgender males, the final size was chosen by comparison with the contralateral testis. Nevertheless, a quarter of cisgender males rated the TP as too small, which may be explained by natural testicular asymmetry, postoperative hematoma or hydrocele, and changes of the native testis over time. In transgender males, the final size was determined intraoperatively by the available neoscrotal tissue, as larger prostheses increase the risk of erosion, while a minimum size is desirable to accommodate a later penile prosthesis pump. This inherently limits achievable implant dimensions, particularly in bilateral reconstruction, and may explain why patient wishes could not always be met. Accordingly, these limitations should be explicitly addressed during preoperative counselling and shared decision-making, with a focus on the balance between aesthetic expectations and surgically feasible outcomes. Furthermore, the perceived firmness may be specific to the device used in this cohort. Alternative prostheses, including all-silicone gel-filled implants from other manufacturers may provide a softer consistency and could be considered in patients concerned about firmness. The higher rate of too firm ratings among cisgender males may also reflect the absence of a reference in transgender males. Most cisgender patients received a unilateral implant and could directly compare it with their native contralateral testis, whereas transgender males had no natural testis as a benchmark for what the consistency feels like.

### Clinical implications and risk factors

From a clinical perspective, the increasing number of transgender patients undergoing gender-affirming surgery highlights the growing relevance of TP implantation in this population [8]. While previous studies have primarily focused on cisgender males following orchiectomy [19, 20], comparative data and data solely including transgender patients remain limited [21]. In this context, the present study contributes to a more comprehensive understanding of outcomes across both groups and supports the integration of standardized patient-reported outcome measures into routine clinical practice. Given the rising demand for gender-affirming procedures, structured preoperative counselling and outcome reporting will be essential to optimize patient selection, manage expectations, and further refine reconstructive strategies. Active smoking was not an exclusion criterion in our cohort. As smoking is a recognized risk factor for wound healing and infection in prosthetic surgery, it should be considered during preoperative counselling and patient selection.

### Limitations

The present study is limited by its retrospective single-center design, which introduces inherent selection bias and limits generalizability. The response rate for PROMs may introduce non-response bias, as individuals with more favourable experiences may be more likely to participate. The cross-sectional assessment of PROMs precludes evaluation of longitudinal changes in satisfaction and regret over time. The relatively small PROM sample, particularly within subgroup analyses, limits statistical power to detect subtle between-group differences. Despite the use of validated instruments, comparison with the broader literature remains constrained by heterogeneous reporting practices and the frequent use of non-validated outcome measures in previous studies. As all patients received prostheses from a single manufacturer, our results may not be transferable to other device types.

## Conclusion

TP implantation yields high surgical satisfaction, low decision regret, and a positive genital self-image in both cisgender and transgender males, supporting its role as a valuable intervention regardless of gender identity. Transgender males face a significantly higher retreatment burden, driven largely by the staged nature of gender-affirming surgery rather than device failure alone, a distinction that must be communicated explicitly during preoperative counselling. Systematic application of validated PROMs remains essential to generate comparable evidence in a field characterized by heterogeneous reporting and will be indispensable to optimize shared decision-making and advance evidence-based care in TP surgery.

## Supplementary Information

Below is the link to the electronic supplementary material.

Supplementary file1 (DOCX 40 KB)

## Data Availability

No datasets were generated or analysed during the current study.
