# Supplementary material for: Testicular prostheses implantation in cisgender and transgender males
Source: World J Urol. 2026 Nov 14;44(1):808. doi: 10.1007/s00345-026-06916-x (PMC13769711; doi:10.1007/s00345-026-06916-x)
Supplement: Supplementary file 1 [file 345_2026_6916_MOESM1_ESM.docx]

Supplementary File 1

Table: Response distributions for individual International Consultation on Incontinence Questionnaire (ICIQ) - Satisfaction items among cisgender and transgender males completing the PROM questionnaire

| ICIQ-Satisfaction | Total  N (%) | Cisgender  N (%) | Transgender  N (%) |
| --- | --- | --- | --- |
| 1. How would you rate the outcome of your surgery? | | | |
| very successful | 27 (63) | 14 (70) | 13 (57) |
| somewhat successful | 13 (30) | 5 (25) | 8 (35) |
| neither successful nor unsuccessful | 2 (4.7) | 0 (0) | 2 (8.7) |
| a little unsuccessful | 1 (2.3) | 1 (5) | 0 (0) |
| very unsuccessful | 0 (0) | 0 (0) | 0 (0) |
| 2. Compared to how you felt before your surgery, how is your condition now? | | | |
| much better | 27 (63) | 8 (40) | 19 (83) |
| a bit better | 3 (7) | 0 (0) | 3 (13) |
| about the same | 12 (28) | 11 (55) | 1 (4.3) |
| a bit worse | 0 (0) | 0 (0) | 0 (0) |
| much worse | 0 (0) | 0 (0) | 0 (0) |
| 3. Would you say you have been able to return to a ‘normal life’ after your surgery? | | | |
| strongly agree | 25 (58) | 9 (45) | 16 (70) |
| agree | 17 (40) | 10 (50) | 7 (30) |
| disagree | 0 (0) | 0 (0) | 0 (0) |
| strongly disagree | 0 (0) | 0 (0) | 0 (0) |
| 4. If you were in the same situation again, would you still have the surgery? | | | |
| yes, definitely | 39 (91) | 20 (100) | 19 (83) |
| yes, probably | 3 (7) | 0 (0) | 3 (13) |
| not sure | 1 (2.3) | 0 (0) | 1 (4.3) |
| no, probably not | 0 (0) | 0 (0) | 0 (0) |
| no, definitely not | 0 (0) | 0 (0) | 0 (0) |
| 5. Would you recommend this surgery to friends or relatives with similar problems? | | | |
| yes, definitely | 31 (72) | 17 (85) | 14 (61) |
| yes, probably | 8 (19) | 2 (10) | 6 (26) |
| not sure | 4 (9.3) | 1 (5) | 3 (13) |
| no, probably not | 0 (0) | 0 (0) | 0 (0) |
| no, definitely not | 0 (0) | 0 (0) | 0 (0) |
| 6. If you had to spend the rest of your life with your symptoms as they are now, how would you feel? | | | |
| perfectly happy | 20 (47) | 9 (45) | 11 (48) |
| somewhat happy | 9 (21) | 4 (20) | 5 (22) |
| mixed feelings | 3 (7) | 1 (5) | 2 (8.7) |
| somewhat unhappy | 1 (2.3) | 0 (0) | 1 (4.3) |
| very unhappy | 4 (9.3) | 1 (5) | 3 (13) |
| desperate | 2 (4.7) | 1 (5) | 1 (4.3) |
| 7. How prepared did you feel for the surgery? | | | |
| completely prepared | 25 (58) | 10 (50) | 15 (65) |
| somewhat prepared | 12 (28) | 6 (30) | 6 (26) |
| somewhat unprepared | 4 (9.3) | 3 (15) | 1 (4.3) |
| completely unprepared | 2 (4.7) | 1 (5) | 1 (4.3) |
| 8. How satisfied were you with any explanation about your surgery? | | | |
| very satisfied | 21 (49) | 10 (50) | 11 (48) |
| quite satisfied | 20 (47) | 9 (45) | 11 (48) |
| quite dissatisfied | 2 (4.7) | 1 (5) | 1 (4.3) |
| very dissatisfied | 0 (0) | 0 (0) | 0 (0) |
| I did not receive any information | 0 (0) | 0 (0) | 0 (0) |
| 9. After the surgery, how much pain did you experience? | | | |
| none | 6 (14) | 4 (20) | 2 (8.7) |
| a little | 29 (67) | 14 (70) | 15 (65) |
| a lot | 6 (14) | 1 (5) | 5 (22) |
| severe pain | 2 (4.7) | 1 (5) | 1 (4.3) |
| b) How much does this bother you? Please ring a number between 0 (not at all) and 10 (a great deal) | | | |
| 0 | 4 (9.3) | 2 (10) | 2 (8.7) |
| 1 | 5 (12) | 2 (10) | 3 (13) |
| 2 | 5 (12) | 3 (15) | 2 (8.7) |
| 3 | 3 (7) | 1 (5) | 2 (8.7) |
| 4 | 3 (7) | 2 (10) | 1 (4.3) |
| 5 | 6 (14) | 2 (10) | 4 (17) |
| 6 | 3 (7) | 1 (5) | 2 (8.7) |
| 7 | 0 (0) | 0 (0) | 0 (0) |
| 8 | 5 (12) | 1 (5) | 4 (17) |
| 9 | 2 (4.7) | 2 (10) | 0 (0) |
| 10 | 1 (2.3) | 0 (0) | 1 (4.3) |
| 10. How satisfied were you with any treatment you had for pain? | | | |
| I did not require pain treatment | 9 (21) | 7 (35) | 2 (8.7) |
| very satisfied | 23 (54) | 7 (35) | 16 (70) |
| quite satisfied | 8 (19) | 4 (20) | 4 (17) |
| quite dissatisfied | 1 (2.3) | 0 (0) | 1 (4.3) |
| very dissatisfied | 1 (2.3) | 1 (5) | 0 (0) |
| 11. How much pain are you experiencing now? | | | |
| none | 35 (81) | 18 (90) | 17 (74) |
| a little | 8 (19) | 2 (10) | 6 (26) |
| a lot | 0 (0) | 0 (0) | 0 (0) |
| severe pain | 0 (0) | 0 (0) | 0 (0) |
| b) How much does this bother you? Please ring a number between 0 (not at all) and 10 (a great deal) | | | |
| 0 | 27 (63) | 14 (70) | 13 (57) |
| 1 | 3 (7) | 1 (5) | 2 (8.7) |
| 2 | 1 (2.3) | 1 (5) | 0 (0) |
| 3 | 1 (2.3) | 0 (0) | 1 (4.3) |
| 4 | 27 (63) | 14 (70) | 13 (57) |
| 5 | 0 (0) | 0 (0) | 0 (0) |
| 6 | 0 (0) | 0 (0) | 0 (0) |
| 7 | 0 (0) | 0 (0) | 0 (0) |
| 8 | 0 (0) | 0 (0) | 0 (0) |
| 9 | 0 (0) | 0 (0) | 0 (0) |
| 10 | 0 (0) | 0 (0) | 0 (0) |
| 12. Were there any complications or side effects with your surgery? | | | |
| no complications | 34 (79) | 18 (90) | 16 (70) |
| yes, minor complications | 7 (16) | 2 (10) | 5 (22) |
| yes, major complications | 1 (2.3) | 0 (0) | 1 (4.3) |
| 13. Overall, has the result of your surgery been…? | | | |
| better than you expected | 13 (30) | 5 (25) | 8 (35) |
| different than expected | 10 (23) | 5 (25) | 5 (22) |
| just as you expected | 18 (42) | 9 (45) | 9 (39) |
| worse than you expected | 1 (2.3) | 0 (0) | 1 (4.3) |
| 14. Overall, how satisfied were you with your surgery? Please ring a number between 0 (not satisfied) and 10 (very satisfied) | | | |
| 0 | 0 (0) | 0 (0) | 0 (0) |
| 1 | 0 (0) | 0 (0) | 0 (0) |
| 2 | 0 (0) | 0 (0) | 0 (0) |
| 3 | 1 (2.3) | 1 (5) | 0 (0) |
| 4 | 0 (0) | 0 (0) | 0 (0) |
| 5 | 2 (4.7) | 1 (5) | 1 (4.3) |
| 6 | 3 (7) | 0 (0) | 3 (13) |
| 7 | 2 (4.7) | 2 (10) | 0 (0) |
| 8 | 6 (14) | 2 (10) | 4 (17) |
| 9 | 13 (30) | 6 (30) | 7 (30) |
| 10 | 11 (26) | 5 (25) | 6 (26) |

*ICIQ-S = International Consultation on Incontinence Questionnaire – Satisfaction; PROM = patient-reported outcome measure*

*Percentages may not add up to 100%, as they are rounded.*

Supplementary File 2

Table: Response distributions for individual Decision Regret Scale (DRS) items among cisgender and transgender males completing the PROM questionnaire

| DRS | Total  N (%) | Cisgender  N (%) | Transgender  N (%) |
| --- | --- | --- | --- |
| 1. It was the right decision | | | |
| strongly agree | 34 (79) | 14 (70) | 20 (87) |
| agree | 8 (19) | 5 (25) | 3 (13) |
| neither agree nor disagree | 1 (2.3) | 1 (5) | 0 (0) |
| disagree | 0 (0) | 0 (0) | 0 (0) |
| strongly disagree | 0 (0) | 0 (0) | 0 (0) |
| 2. I regret the choice that was made | | | |
| strongly agree | 1 (2.3) | 1 (5) | 0 (0) |
| agree | 0 (0) | 0 (0) | 0 (0) |
| neither agree nor disagree | 1 (2.3) | 1 (5) | 0 (0) |
| disagree | 6 (14) | 2 (10) | 4 (17) |
| strongly disagree | 35 (81) | 16 (80) | 19 (83) |
| 3. I would go for the same choice if I had to do it over again | | | |
| strongly agree | 33 (77) | 13 (65) | 20 (87) |
| agree | 8 (19) | 5 (25) | 3 (13) |
| neither agree nor disagree | 0 (0) | 0 (0) | 0 (0) |
| disagree | 1 (2.3) | 1 (5) | 0 (0) |
| strongly disagree | 0 (0) | 0 (0) | 0 (0) |
| 4. The choice did me a lot of harm | | | |
| strongly agree | 0 (0) | 0 (0) | 0 (0) |
| agree | 0 (0) | 0 (0) | 0 (0) |
| neither agree nor disagree | 1 (2.3) | 1 (5) | 0 (0) |
| disagree | 6 (14) | 5 (25) | 1 (4.3) |
| strongly disagree | 36 (84) | 14 (70) | 22 (96) |
| 5. The decision was a wise one | | | |
| strongly agree | 31 (72) | 12 (60) | 19 (83) |
| agree | 10 (23) | 6 (30) | 4 (17) |
| neither agree nor disagree | 2 (4.7) | 2 (10) | 0 (0) |
| disagree | 0 (0) | 0 (0) | 0 (0) |
| strongly disagree | 0 (0) | 0 (0) | 0 (0) |

*DRS = Decision Regret Scale; PROM = patient-reported outcome measure*

*Percentages may not add up to 100%, as they are rounded.*

Supplementary File 3

Table: Response distributions for individual Male Genital Self-Image Scale (MGSIS) items among cisgender and transgender males completing the PROM questionnaire

| MGSIS | Total  N (%) | Cisgender  N (%) | Transgender  N (%) |
| --- | --- | --- | --- |
| 1. I feel positively about my genitals | | | |
| strongly disagree | 0 (0) | 0 (0) | 0 (0) |
| disagree | 1 (2.3) | 1 (5) | 0 (0) |
| agree | 29 (67) | 12 (60) | 17 (74) |
| strongly agree | 13 (30) | 7 (35) | 6 (26) |
| 2. I am satisfied with the appearance of my genitals | | | |
| strongly disagree | 0 (0) | 0 (0) | 0 (0) |
| disagree | 6 (14) | 2 (10) | 4 (17) |
| agree | 31 (72) | 14 (70) | 17 (74) |
| strongly agree | 6 (14) | 4 (20) | 2 (8.7) |
| 3. I feel comfortable letting a sexual partner look at my genitals | | | |
| strongly disagree | 0 (0) | 0 (0) | 0 (0) |
| disagree | 3 (7) | 0 (0) | 3 (13) |
| agree | 20 (47) | 10 (50) | 10 (44) |
| strongly agree | 20 (47) | 10 (50) | 10 (44) |
| 4. I am satisfied with the size of my genitals | | | |
| strongly disagree | 1 (2.3) | 1 (5) | 0 (0) |
| disagree | 9 (21) | 2 (10) | 7 (30) |
| agree | 22 (51) | 12 (60) | 10 (44) |
| strongly agree | 11 (26) | 5 (25) | 6 (26) |
| 5. I think my genitals work the way they are supposed to work | | | |
| strongly disagree | 0 (0) | 0 (0) | 0 (0) |
| disagree | 8 (19) | 2 (10) | 6 (26) |
| agree | 19 (44) | 9 (45) | 10 (44) |
| strongly agree | 16 (37) | 9 (45) | 7 (30) |
| 6. I feel comfortable letting a healthcare provider examine my genitals | | | |
| strongly disagree | 0 (0) | 0 (0) | 0 (0) |
| disagree | 3 (7) | 0 (0) | 3 (13) |
| agree | 19 (44) | 11 (55) | 8 (35) |
| strongly agree | 21 (49) | 9 (45) | 12 (52) |
| 7. I am not embarrassed about my genitals | | | |
| strongly disagree | 0 (0) | 0 (0) | 0 (0) |
| disagree | 11 (26) | 3 (15) | 8 (35) |
| agree | 17 (40) | 9 (45) | 8 (35) |
| strongly agree | 15 (35) | 8 (40) | 7 (30) |

*MGSIS = Male Genital Self-Image Scale; PROM = patient-reported outcome measure*

*Percentages may not add up to 100%, as they are rounded.*

Supplementary File 4

Table: Patient-reported contextual factors among cisgender and transgender males completing the PROM questionnaire

| Variable | Total  (N=43) | Cisgender  (N=20) | Transgender  (N=23) | p-value |
| --- | --- | --- | --- | --- |
| Partner at implantation, N (%) | | | | 0.002 |
| No | 26 (61) | 7 (35) | 19 (83) |  |
| Yes | 17 (40) | 13 (65) | 4 (17) |  |
| Importance of being offered a TP, N (%) | | | | 0.9 |
| Very unimportant | 0 (0) | 0 (0) | 0 (0) |  |
| Unimportant | 0 (0) | 0 (0) | 0 (0) |  |
| Partly/Partly | 2 (4.7) | 1 (5) | 1 (4.3) |  |
| Rather important | 7 (16) | 3 (15) | 4 (17) |  |
| Very important | 34 (79) | 16 (80) | 18 (78) |  |
| Reason for choosing a TP (multiple answers allowed), N (%) | | | |  |
| Aesthetic reasons | 23 (54) | 13 (65) | 10 (44) | 0.2 |
| Increased self-confidence | 13 (30) | 5 (25) | 8 (35) | 0.5 |
| Improved body image | 20 (47) | 6 (30) | 14 (61) | 0.067 |
| Feeling of completeness | 38 (88) | 17 (85) | 21 (91) | 0.7 |
| Impact on partnership or sexual life | 8 (19) | 3 (15) | 5 (22) | 0.7 |
| Preference for preoperative discussion of TP, N (%) | | | | <0.001 |
| No | 12 (28) | 2 (10) | 10 (44) |  |
| Yes | 22 (51) | 16 (80) | 6 (26) |  |
| Unsure | 6 (14) | 0 (0) | 6 (26) |  |
| Not reported | 3 (6.9) | 2 (10) | 1 (4.3) |  |
| Testosterone replacement therapy, N (%) | | | | <0.001 |
| Never | 17 (40) | 16 (80) | 1 (4.3) |  |
| Former | 1 (2.3) | 0 (0) | 1 (4.3) |  |
| Current | 25 (58) | 4 (20) | 21 (91) |  |
| Highest education level, N (%) | | | | 0.009 |
| Completed vocational training | 15 (35) | 4 (20) | 11 (48) |  |
| Higher education entrance qualification | 7 (16) | 1 (5) | 6 (26) |  |
| University degree | 20 (47) | 14 (70) | 6 (26) |  |
| Not reported | 1 (2.3) | 1 (0.5) | 0 (0) |  |

*PROM = patient-reported outcome measure; TP = Testicular Prostheses*

*Percentages may not add up to 100%, as they are rounded.*
